# MRI Evaluation of Cervical, Spring, and Interosseous Talocalcaneal Ligament Orientation in Progressive Collapsing Foot Deformity

**DOI:** 10.1177/10711007251363927

**Published:** 2025-09-23

**Authors:** Alexander Chang, Brady Huang, Ian Foran

**Affiliations:** 1 University of California, San Diego, USA

**Keywords:** Progressive collapsing flatfoot deformity, acquired adult flatfoot deformity, PCFD, AAFD, MRI, x-ray, cervical ligament, interosseous talocalcaneal ligament, spring ligament, flatfoot, radiographic analysis

## Abstract

**Background:**

Progressive collapsing foot deformity (PCFD) is a complex condition characterized by progressive ligamentous and osseous changes in the hindfoot, midfoot, and forefoot. Although osseous changes at the subtalar and transverse tarsal joints have been well studied, ligamentous anatomy in PCFD is less understood. This study evaluates the cervical, interosseous talocalcaneal, and superomedial fibers of the spring ligament in patients with PCFD vs controls using magnetic resonance imaging (MRI) analysis.

**Methods:**

Nonweightbearing MRI and weightbearing radiographs of 39 patients (23 PCFD, 16 controls) were retrospectively reviewed. MRIs measured the coronal plane orientation of the interosseous talocalcaneal, cervical, and superomedial spring ligaments relative to the subtalar joint middle facet. Radiographic data included anteroposterior (AP) talonavicular coverage percentage, AP talocalcaneal angle (Kite), lateral talar–first metatarsal angle (Meary), talar declination angle, and calcaneal pitch. Two observers measured each radiographic and MRI angle. Statistical analysis included an independent *t* test and intraclass correlation coefficient (ICC) to assess interobserver reliability.

**Results:**

PCFD patients demonstrated significantly more horizontal ligament orientations than controls, with reduced cervical (25.5 vs 45 degrees, *P* < .001), superomedial spring (11.5 vs 23.1 degrees, *P* < .001), and interosseous talocalcaneal ligament angles (39.5 vs 49.0 degrees, *P* = .005). Radiographically, PCFD patients had decreased talonavicular coverage (64.5% vs 80.9%, *P* < .001), increased Meary angle (22.2 vs −2.3 degrees, *P* < .001), increased talar declination (37.0 vs 20.6 degrees, *P* < .001), increased Kite angle (20.7 vs 17.2 degrees, *P* = .079), and decreased calcaneal pitch (15.5 vs 24.6 degrees, *P* < .001). Interobserver reliability was excellent, with ICC values exceeding 0.94 for all measurements except interosseous talocalcaneal ligament angle (ICC = 0.83).

**Conclusion:**

On nonweightbearing MRI, PCFD patients showed more horizontal orientation of key subtalar ligaments than controls; whether these differences persist under physiologic load should be confirmed with weightbearing imaging.

## Graphical abstract

**Graphical Abstract:**
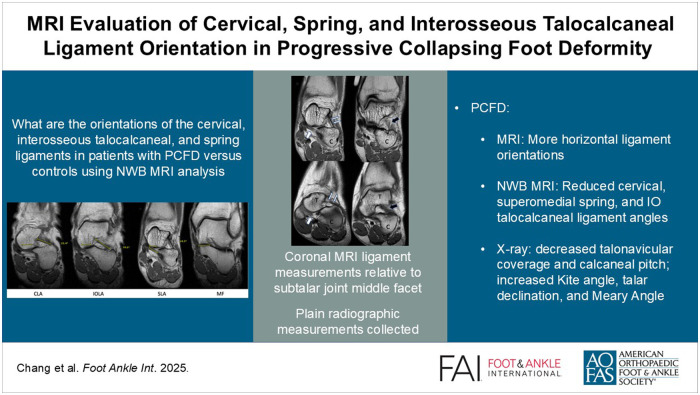
This is a visual representation of the abstract.

**Level of Evidence:** Level III, retrospective comparative study.

## Introduction

Progressive collapsing foot deformity (PCFD) is a complex and often debilitating condition that is estimated to affect up to 5 million Americans.^2,10^ The etiology remains unknown, and there is debate regarding whether bony, ligamentous, or tendinous pathology—or some combination thereof—is causal. Regardless, it is understood that this condition progressively affects nearly all joints in the foot, leading to the classic bony alterations of peritalar subluxation, subfibular impingement, hindfoot valgus, midfoot abduction, and forefoot supination (varus).^2,9,15,20,25^

The general osseous changes in PCFD are better understood. These are exhibited in radiographic changes in talar declination, calcaneal pitch, talar-1st metatarsal (Meary) angle, talonavicular uncoverage, and talocalcaneal (Kite) angle.^15,17,21,25,30^ In contrast, the complex ligamentous changes in PCFD are not as well understood.^2,15^ The majority of recent imaging analysis literature on PCFD has concentrated on assessing osseous and structural changes through plain radiographs and weightbearing computed tomography (WBCT).^1,5,12,25,30^ Recently, more attention has been given to subtalar ligamentous insufficiency in PCFD, particularly the role of the cervical ligament.^1,5,9,12,25^ Current literature has not thoroughly assessed the pathoanatomic changes and relationships of the cervical, spring, and interosseous talocalcaneal ligaments in PCFD compared to a control group using magnetic resonance imaging (MRI).

This study aims to further evaluate the osseous and ligamentous pathoanatomic alterations in PCFD using plain radiographs and MRI analysis, specifically focusing on the cervical, superomedial spring, and interosseous talocalcaneal ligaments.

## Methods

Following institutional review board (IRB) approval, a retrospective review of medical records and imaging was performed at a single academic institution. Between April 2010 and November 2024, records were identified and collected for screening into the control group, whereas records from April 2014 to April 2021 were assessed for inclusion in the PCFD group. Patients with PCFD were identified by diagnosis of “valgus,” “pes planus,” “adult acquired flatfoot deformity,” or “posterior tibial tendon dysfunction.” The diagnosis of PCFD was confirmed by review of radiographic parameters and chart review. Control patients were identified by reviewing nonweightbearing ankle MRIs of patients that (1) had no diagnosis of PCFD as noted above, (2) had an MRI musculoskeletal-radiologist impression that identified no ankle or hindfoot pathology, and (3) no signs of flatfoot morphology on weightbearing radiographs. Additional inclusion criteria included being at least 18 years of age, having ankle MRIs that were of at least 1.5-tesla (T) strength and of sufficient quality for review, and having weightbearing radiographs of the foot within 1 year of the MRI. MRI examinations were performed on a variety of platforms, including GE 1.5-T Signa HDxt, GE 3-T MR750 Signa Premier, GE 3-T Signa HDxt, Siemens 1.5-T Magnetom Aera. Although there were different vendor platforms, the following sequences were common to all examinations: axial T1-weighted (time to response [TR] range / time to echo [TE] range: 400-800/10-15 milliseconds [ms]), axial proton density (PD)-weighted fat-suppressed (1600-2800/20-40 ms), coronal T1-weighted (400-800/10-15 ms), coronal T2-weighted fat-suppressed (3000-8000/50-90 ms), sagittal T1-weighted (400-800/10-15 ms), sagittal T2-weighted fat-suppressed (3000-8000/50-90 ms), and PD-weighted coronal oblique fat-suppressed (4000-5000/15-20). Acquisition matrix ranged from 320-384 × 224-288 pixels, and slice thickness was 3 mm, with an interslice gap of 0.5 mm. The quality of the MRI was assessed subjectively by both readers if the ligaments and osseous structures could be identified clearly, and there were no significant artifacts that would hamper measurements (ie, patient motion, metallic susceptibility, and incomplete examinations). Exclusion criteria included patients with foot masses, tumors, coalitions, ankle or hindfoot arthritis, previous midfoot or hindfoot surgery that pre-dated imaging, and prior major hindfoot and midfoot fractures.

Patient demographic data at the time of imaging were collected, including sex, body mass index (BMI), and age, from the institution’s electronic medical record system (Epic Systems Corporation, Verona, WI).

**Figure 1.**
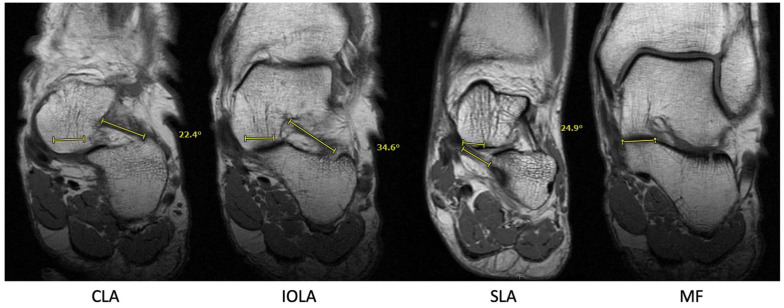
Coronal T1-weighted magnetic resonance images in a control patient demonstrating cervical ligament angle (CLA), interosseous talocalcaneal ligament angle (IOA), and spring ligament angle (SLA). All measurements were made with reference to a line drawn in parallel with the medial facet (MF).

Radiographic data were collected from weightbearing radiographs, including anteroposterior (AP) talonavicular coverage percentage, AP talocalcaneal angle (Kite angle), lateral talar–first metatarsal angle (Meary angle), talar declination angle, and calcaneal pitch. MRIs with standard clinical imaging protocol, including T1-weighted coronal plane sequences, were used to measure the interosseous (IO) talocalcaneal ligament angle, the cervical ligament angle, and the spring ligament (specifically the superomedial calcaneonavicular fibers) angle relative to the subtalar joint middle facets (Figure 1).^18^ T1-weighted images were used as the low signal intensity ligaments best contrasted with the background of hyperintense surrounding adipose tissue in the tarsal sinus and canal. Measurements were not performed using T2-weighted or PD-weighted images, as these were all performed with fat suppression, resulting in loss of contrast between the ligaments and the adipose tissue. Similar investigations have used either T1 or T2 coronal sequences without fat suppression to best visualize the ligaments, which similarly allow identification of the ligaments from background adipose tissue.^3,14^ As described by previous authors, the cervical ligament could be identified in the anterior part of the tarsal sinus, attaching superiorly from a small tubercle on the inferolateral talar neck and onto the dorsal surface of the calcaneus, whereas the interosseous talocalcaneal ligament could be visualized running obliquely from the talus to the calcaneus inferiorly within the tarsal sinus.^3,14^ The MRI appearance of the superomedial calcaneonavicular component of the spring ligament complex has also been previously described on MRI with cadaveric correlation, having a broad attachment on the anteromedial margin of the middle facet of the sustentaculum tali and attaching to the superomedial navicular.^18^ Measurements were performed using Sectra IDS7 viewing software (version 26.2; Linköping, Sweden). The angles were measured as follows: (1) the best coronal slice of the middle subtalar facet joint at its most horizontal orientation was chosen as a landmark; (2) the coronal slice with the ligament best visualized was chosen for measurement; and (3) a line was drawn parallel to the middle facets, and a line was drawn parallel to the orientation of each ligament using the imaging software’s dedicated angle measurement function, which enabled assessment across multiple MRI slices. The angle subtending these lines constituted the respective ligament angles. Two independent observers (an orthopaedic surgical resident and orthopaedic fellowship-trained foot and ankle attending surgeon with 5 years of experience) measured all radiographic and MRI angles. Observers could not be blinded to the patient group when making measurements, as patients with PCFD morphology were obvious from basic observation, which was required to make measurements.

The mean of each radiographic and MRI measurement was collected in both the PCFD and control group. Univariate analysis comparing angles and radiographic parameters between the PCFD and control group was performed using the independent student *t* test. A Pearson correlation coefficient analysis was used to measure the association between our radiographic and MRI measurements. To evaluate our Pearson correlation coefficients, an *r* value, either positive or negative, from 0.90 to 1 was considered very strong correlation, 0.7 to 0.89 a strong correlation, 0.40 to 0.69 a moderate correlation, 0.10 to 0.39 a weak correlation, and 0.0 to 0.1 a negligible correlation.^17,21,27^ To assess agreement between the measurements of each observer for each angle category, interobserver reliability was tested using the intraclass correlation coefficient (ICC) and a 95% CI.^28^ To evaluate our ICC, a value within 0.81 to 1.0 was considered very good, 0.61 to 0.8 good, and 0.41 to 0.6 moderate.^12^ A post hoc power analysis was performed. All data analyses were performed using IBM SPSS Statistics (version 30).

## Results

Records of 144 patient records were reviewed for potential inclusion in this retrospective study. Of these, 72 were evaluated as potential controls, with 16 meeting final inclusion criteria. The remaining 56 were excluded because of inadequate radiographs (nonweightbearing or ankle radiograph; n = 48) and excessive time between radiograph and MRI (n = 8). The other 72 patients were assessed for potential inclusion in the PCFD group, with 23 ultimately meeting criteria. Forty-nine were excluded for the following reasons: inadequate radiographs (nonweightbearing or ankle radiograph; n = 13); excessive time between radiograph and MRI (n = 1); diagnosis not consistent with PCFD (n = 11); no MRI available (n = 3); MRI of insufficient magnet strength (<1.5 tesla; n = 1); prior midfoot or hindfoot surgery (n = 13); abnormal foot morphology including masses, tumors, or coalitions (n = 5); and prior major fractures (n = 2). Thirty-nine patients fit our criteria, with 23 (11 female, 12 male) patients in the PCFD group and 16 patients (12 female, 4 male) in the control group. The average age of patients in the PCFD group was 53.3 (range, 19-80) years and 45.6 (range, 20-80) years in the control group (Table 1). The average BMI in the PCFD and control group was 28.3 and 23.3, respectively. Age and gender were not found to have a statistically significant difference (*P* = .202 and *P* = .087, respectively), whereas BMI was found to have a statistically significant difference (*P* < .001) between our PCFD and control group.

**Table 1.** Demographic Data of the Total Study Population.^a^

	PCFD	Control	*P* Value
Number of patients	23	16	
Age, y, mean ± SD (range)	53.3 ± 16.5 (19-80)	45.6 ± 22.5 (20-80)	.202
BMI, mean ± SD	28.3 ± 4.0	23.3 ± 3.5	**<.001**
Gender, female/male, n	11:12	12:4	.087

Abbreviations: BMI, body mass index; PCFD, progressive collapsing foot deformity.

a Bolded values indicate statistical significance.

**Figure 2.**
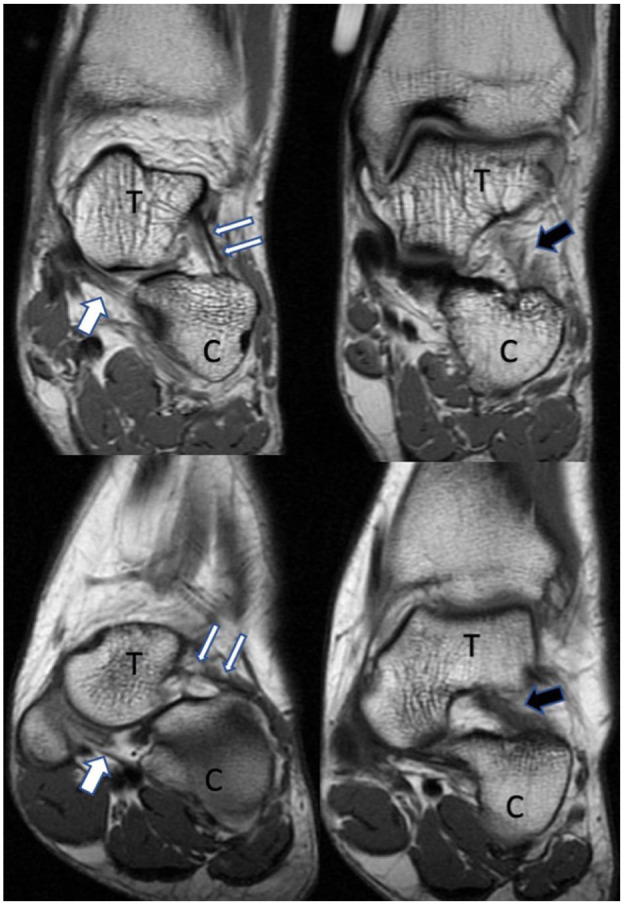
Coronal T1-wighted magnetic resonance images in a control patient (top images) and a PCFD patient (bottom images). Images highlight the cervical ligament (thin white arrows), spring ligament (thick white arrow), and interosseous talocalcaneal ligament (thick black arrow). Note increased horizontal orientation of the cervical and interosseous talocalcaneal ligaments in the PFCD patient compared with the control patient. C, calcaneus; PCFD, progressive collapsing foot deformity; T, talus.

The average measurements of each radiographic and MRI angle were notably different between the PCFD and control groups (Table 2). PCFD patients demonstrated significantly more horizontal ligament orientations than controls, with reduced cervical (25.5 vs 45 degrees, *P* < .001), spring (11.5 vs 23.1 degrees, *P* < .001), and interosseous talocalcaneal ligament angles (39.5 vs 49.0 degrees, *P* = .005) (Figure 2).

**Table 2.** Mean Angles With SDs for Each Measurement by Each Observer in Both PCFD and Control Group.^a^

	PCFD	Control	*P* Value
Talonavicular coverage (%)	64.5 ± 10.3	80.9 ± 7.8	**<.001**
Kite angle	20.7 ± 7.0	17.2 ± 8.3	.079
Meary angle	22.2 ± 11.0	−2.3 ± 4.6	**<.001**
Talar declination	37.0 ± 9.4	20.6 ± 3.3	**<.001**
Calcaneal pitch	15.5 ± 4.7	24.6 ± 5.5	**<.001**
IO talocalcaneal ligament angle	39.5 ± 10.4	49.0 ± 11.0	**.005**
Cervical ligament angle	25.5 ± 11.2	45.0 ± 13.9	**<.001**
Spring ligament angle	11.5 ± 5.7	23.1 ± 9.8	**<.001**

Abbreviations: BMI, body mass index; PCFD, progressive collapsing foot deformity.

a Bolded values indicate statistical significance.

Radiographically, PCFD patients had decreased talonavicular coverage (64.5% vs 80.9%, *P* < .001), increased Meary angle (22.2 vs −2.3 degrees, *P* < .001), increased talar declination (37.0 vs 20.6 degrees, *P* < .001), and decreased calcaneal pitch (15.5 vs 24.6 degrees, *P* < .001). Kite angle was increased in PCFD patients but did not reach statistical significance (20.7 degrees vs 17.2 degrees, *P* < .079).

The Pearson correlation coefficients for the cervical ligament fell within the moderate correlation category (*r* = 0.4-0.69) for all radiographic measurements, including talonavicular coverage percentage, Meary angle, talar declination, and calcaneal pitch, except for Kite angle, which exhibited a weak correlation category (0.1-0.39). The positive coefficient values demonstrate that as talonavicular coverage or calcaneal pitch decreases, so does the cervical ligament angle (Table 3).

**Table 3.** Pearson Correlation Coefficients With Associated *P* Values.^a^

	IO Talocalcaneal Ligament Angle (*P* Value)	Cervical Ligament Angle (*P* Value)	Spring Ligament Angle (*P* Value)
Talonavicular coverage (%)	0.188 (.252)	0.433 **(.006)**	0.443 **(.005)**
Kite angle	−0.251 (.123)	−0.334 **(.038)**	−0.110 (.506)
Meary angle	−0.356 (.026)	−0.624 **(<.001)**	−0.484 **(.002)**
Talar declination	−0.410 **(.01)**	−0.580 **(<.001)**	−0.430 **(.006)**
Calcaneal pitch	0.330 **(.04)**	0.481 **(.002)**	0.397 **(.012)**

Abbreviation: IO, interosseus.

a Bolded values indicate statistical significance.

Moderate correlations for the spring ligament were observed in the talonavicular coverage percentage, Meary angle, and talar declination, with the same direct and inverse relationships described for the cervical ligament. Regarding the interosseous talocalcaneal ligament angle, only talar declination met criteria for moderate correlation, demonstrating increases in talar declination angle correlating with decreasing IO talocalcaneal ligament angles (Table 3).

All intraclass correlation coefficients (ICCs) were higher than 0.94, except for the interosseous talocalcaneal ligament angle (ICC = 0.83), which remained above the ICC 0.81 threshold to be considered in the highest category of “very good” (Table 4).

**Table 4.** Intraclass Correlation Coefficients for Evaluating Interobserver Reliability.

	Intraclass Correlation Coefficient (95% CI)
Talonavicular coverage %	0.95 (0.91-0.97)
Kite angle	0.94 (0.89-0.97
Meary angle	0.99 (0.98-0.99)
Talar declination	0.96 (0.93-0.98)
Calcaneal pitch	0.99 (0.99-0.99)
IO talocalcaneal ligament angle	0.83 (0.67-0.91)
Cervical ligament angle	0.94 (0.89-0.97)
Spring ligament angle	0.94 (0.88-0.97)

Abbreviation: IO, interosseus.

## Discussion

Despite being a relatively common condition, the pathology and etiology of PCFD remain incompletely understood. The condition has been challenging to study due to the numerous joints involved and the variability of presentation. MRI and weightbearing radiographs of 39 patients (23 PCFD, 16 controls) were retrospectively reviewed. Weightbearing CT (WBCT) has provided a leap forward in our understanding of the bony anatomy of this condition, especially as it relates to the subtalar joint.^1,5,10,13,22,23,30^ More recently, there has been increasing interest in subtalar ligamentous morphology and the possible role for reconstruction of these ligaments in surgical management.^16^ Furthermore, some of the ligament orientations we observed may reflect developmental morphology rather than acquired attenuation, a distinction our nonweightbearing MRI protocol cannot resolve.

To our knowledge, this is the first study to compare MRI ligament morphology in PCFD patients vs control subjects. Our study demonstrates that the IO talocalcaneal, cervical, and spring ligaments all assume a more horizontal orientation in PCFD compared with controls. Additionally, we found correlation between a more horizontal orientation of these ligaments and radiographic parameters associated with PCFD. It should be appreciated that although our results show a different ligament morphology in PCFD patients vs controls, this study does not assess the functional competence of these ligaments, nor does it indicate ligament pathology. Our study also does not determine whether these morphologic parameters are developmental or acquired. Nonetheless, differences in orientation may reasonably be expected to result in altered function of the ligaments and their length-tension parameters.

Our study demonstrated excellent interobserver reliability with nearly all measurements above an ICC of 0.94. The only exception was the interosseous talocalcaneal angle, with an ICC of 0.83. Notably, an ICC higher than 0.81 is classified in the highest category of reliability.^28,29^ This high level of reliability suggests that measurement of these ligament angles is highly reproducible. No special sequencing was necessary, suggesting that these measurements can be made using standard MRI techniques.

Our findings add to those of Kim et al,^12^ who demonstrated that attenuation of the cervical ligament was associated with increased talonavicular subluxation in the axial plane, and tearing of the cervical ligament was correlated with increased abduction deformity of the foot and medial arch collapse. Deland et al^6^ found similar findings of cervical ligament degeneration in a significant number of PCFD patients. Kim et al^14^ and Deland et al^6^ both defined ligament pathology based on a subjectively graded classification system looking at signal intensity and morphology of the ligaments. Their findings may complement our finding that increased horizontal orientation of the cervical ligament more strongly correlated with radiographic parameters of PCFD compared to the IO talocalcaneal or spring ligaments. Although we did not observe any fully “torn” ligaments in our study, it is possible that the finding of increased horizontal inclination of the ligaments corresponds to ligament attenuation seen in their study. Previous investigations of the knee cruciate ligaments have shown that plastic or stretching deformation may occur without apparent ligament fiber discontinuity on MRI. It is possible that a similar phenomenon is occurring within the talocalcaneal ligaments whereby ligament continuity is observed in the setting of functionally incompetent ligaments, and therefore, ligament continuity should not be equated with ligament integrity.^24,26^

Treatment of flexible PCFD has traditionally focused on multijoint extraarticular correction of the deformity through osteotomies and posterior tibial tendon reconstruction.^19,21,25,27^ Some limited research has supported spring ligament reconstruction.^7,31^ However, there has been comparatively little research into the potential role for subtalar ligament reconstruction in PCFD, with no studies to our knowledge evaluating subtalar ligament reconstruction in this population. Femino et al^8^ demonstrated that cervical ligament transection produced talonavicular joint collapse after prior transection of the deltoid, spring, and IO ligaments in a PCFD cadaveric model. There have been several studies evaluating subtalar ligament reconstruction in the setting of subtalar instability.^4,11^ Given the abnormalities of the subtalar ligaments as demonstrated by our study and others, we echo the call by Kim et al^12^ for further research into a potential role for subtalar ligament reconstruction in PCFD.

Our study has several limitations. MRI examinations were conducted nonweightbearing, with the patient’s feet artificially positioned within an extremity coil during imaging. It is well-known that weightbearing significantly alters bony alignment. This potentially limits the utility of our measurements in determining “normal” ligament orientation for reconstruction. Further study is needed to see how ligament morphology changes in simulated weightbearing MRI, both in controls and in PCFD patients. Because of differences in the morphology of these ligaments, determining which coronal MRI slices to use for measurements was somewhat subjective. Furthermore, there may be differences in osseous morphology that may also impact measurements. The middle facets of the talus and calcaneus may be sloped and also affect angle measurements. However, the use of a consistent measurement protocol, along with our excellent interobserver reliability, suggests that the measurements are reproducible.

Additionally, we had a limited number of PCFD and control patients in the study which may limit the generalizability and external validity of our findings. The small sample size also yields wide CIs around several key estimates, and the study was not prospectively powered to detect modest but clinically important differences. Given the variability in morphology of PCFD patients, it is certainly possible that certain PCFD subgroups will not have the same alterations in ligament orientation. It is also possible that our “control” patients did not represent normal foot conditions, although MRI and radiographic parameters did not demonstrate any abnormality. Because control MRIs were derived from patients with clinical indications for imaging, selection bias may have influenced the patient composition, limiting its representation of a truly asymptomatic or healthy population. Observers could not be blinded to the control vs PCFD group because of the frankly different morphology between the different groups, which was actively being measured. This could have introduced bias. Body mass index (BMI) was slightly different between the 2 groups, which may have been a confounding variable. Elevated BMI may place increased mechanical stress on foot and ankle structures, potentially altering alignment, ligament orientation, and angle measurements. Consequently, the higher BMI observed in the PCFD group introduces the possibility that some of the observed structural differences may be attributable to BMI, potentially confounding the comparisons between groups.

## Conclusion

Patients with PCFD demonstrate significantly more horizontal orientation of the cervical, superomedial spring, and interosseous talocalcaneal ligaments compared to controls on nonweightbearing MRI; however, the clinical significance of these exploratory findings remains uncertain and should be confirmed in larger, weightbearing imaging studies.

## Supplemental Material

sj-pdf-1-fai-10.1177_10711007251363927 – Supplemental material for MRI Evaluation of Cervical, Spring, and Interosseous Talocalcaneal Ligament Orientation in Progressive Collapsing Foot Deformity Supplemental material, sj-pdf-1-fai-10.1177_10711007251363927 for MRI Evaluation of Cervical, Spring, and Interosseous Talocalcaneal Ligament Orientation in Progressive Collapsing Foot Deformity by Alexander Chang, Brady Huang and Ian Foran in Foot & Ankle International
